# DOPA-Substituted KLVFFA Peptides Potently Inhibit Amyloid-β Aggregation and Mitigate Neurotoxicity

**DOI:** 10.3390/ijms27219503

**Published:** 2026-10-24

**Authors:** Kiyoshi Fukuhara, Kazunori Mori, Yoshio Okiyama, Takashi Takaki, Takashi Misawa, Keisuke Tsuchiya, Yosuke Demizu, Motoko Shibanuma, Akiko Ohno

**Affiliations:** 1 Division of Organic and Medicinal Chemistry, Department of Pharmaceutical Sciences, School of Pharmacy, Showa Medical University, 1-5-8 Hatanodai, Shinagawa-ku, Tokyo 142-8555, Japan; 2 Division of Cancer Cell Biology, Department of Pharmaceutical Sciences, School of Pharmacy, Showa Medical University, 1-5-8 Hatanodai, Shinagawa-ku, Tokyo 142-8555, Japan; mori@pharm.showa-u.ac.jp (K.M.); smotoko@pharm.showa-u.ac.jp (M.S.); 3 Division of Biological Chemistry, Department of Pharmaceutical Sciences, School of Pharmacy, Showa Medical University, 1-5-8 Hatanodai, Shinagawa-ku, Tokyo 142-8555, Japan; 4 Graduate School of Human Development and Environment, Kobe University, 3-11 Tsurukabuto, Nada-ku, Kobe 657-8501, Japan; okiyama.yoshio@gmail.com; 5 Center for Electron Microscopy, Showa Medical University, 1-5-8 Hatanodai, Shinagawa-ku, Tokyo 142-8555, Japan; takaki@med.showa-u.ac.jp; 6 Division of Organic Chemistry, National Institute of Health Sciences, 3-25-26 Tonomachi, Kawasaki-ku, Kawasaki City 210-9501, Japan; misawa@nihs.go.jp (T.M.); demizu@nihs.go.jp (Y.D.); 7 Division of Drugs, National Institute of Health Sciences, 3-25-26 Tonomachi, Kawasaki-ku, Kawasaki City 210-9501, Japan; keisuke-tsuchiya@nihs.go.jp; 8 Division of Genome Safety Science, National Institute of Health Sciences, 3-25-26 Tonomachi, Kawasaki-ku, Kawasaki City 210-9501, Japan

**Keywords:** Alzheimer’s disease, amyloid-β, aggregation, KLVFFA, neurotoxicity

## Abstract

Alzheimer’s disease (AD) is driven primarily by the self-assembly of amyloid-β (Aβ) peptides, a process stabilized by intermolecular π–π stacking interactions involving Phe19 and Phe20 residues of adjacent Aβ molecules. To competitively disrupt these interactions, we designed novel peptide inhibitors derived from the Aβ recognition motif KLVFFA (Aβ_16–21_), in which phenylalanine residues were replaced with L-3,4-dihydroxyphenylalanine (DOPA), featuring a catechol moiety capable of enhanced aromatic stacking and hydrogen bonding. The DOPA-substituted peptides effectively inhibited Aβ_1–42_ (Aβ42) aggregation, as demonstrated via Thioflavin T fluorescence assays, circular dichroism spectroscopy, and transmission electron microscopy. These complementary analyses collectively revealed substantial suppression of β-sheet formation and fibril elongation. Notably, the anti-aggregation potency was dependent on the substitution position, with the Phe19-substituted analog (KDF) exhibiting stronger inhibitory activity than the Phe20-substituted analog (KFD). The dual-substituted analog (KDD) displayed the greatest overall anti-aggregation activity. Molecular simulations further supported the superior activity of KDF over KFD, revealing a more favorable binding free energy for KDF compared to the parent peptide. Furthermore, the DOPA peptides potently mitigated Aβ42-induced neurotoxicity in SH−SY5Y cells, with the dual-substituted analog (KDD) providing the highest level of cytoprotection. Collectively, these findings validate that targeted modulation of aromatic interactions through DOPA substitution represents a powerful molecular strategy for controlling Aβ42 self-assembly, positioning these peptides as promising therapeutic tools for the treatment of AD.

## 1. Introduction

Alzheimer’s disease (AD) is the most prevalent neurodegenerative disorder, accounting for approximately 60–70% of all dementia cases worldwide. Clinically, AD manifests as progressive impairments in memory, orientation, and executive judgment, accompanied by personality and behavioral changes that ultimately compromise activities of daily living [1,2]. Neuropathologically, widespread synaptic loss and neuronal degeneration result from the aberrant accumulation of misfolded proteins, resulting in irreversible cognitive decline [3]. According to the amyloid cascade hypothesis, the pathological deposition of amyloid-β (Aβ) peptides is a central initiating factor in AD onset and progression [4]. Aβ is generated by sequential cleavage of the amyloid precursor protein by β-secretase and γ-secretase. Under physiological conditions, a dynamic equilibrium between Aβ production and clearance prevents pathological accumulation. However, disruption of this balance promotes self-assembly through a multistep aggregation pathway, resulting in progression from soluble oligomers to mature fibrillar amyloids [5]. Emerging evidence implicates soluble oligomers as particularly neurotoxic species, as they disrupt membrane integrity, impair receptor-mediated signaling, perturb Ca^2+^ homeostasis, and increase the generation of reactive oxygen species (ROS). These effects, in turn, promote tau hyperphosphorylation and neurofibrillary tangle formation [6,7,8].

Given its central etiological role, inhibiting Aβ_1–42_ (Aβ42) aggregation is a promising therapeutic strategy. Several classes of agents, including monoclonal antibodies, small molecules, and natural products, are currently under investigation for this purpose. Anti-Aβ antibodies such as lecanemab and donanemab have demonstrated clinically meaningful efficacy by reducing the brain amyloid burden [9,10]. Among small molecules, transthyretin stabilizers and compounds such as tolfenamic acid suppress oligomer formation by interfering with key aggregation interfaces [11,12]. Additionally, polyphenolic natural products, including resveratrol and curcumin, have been reported to attenuate β-sheet assembly through π–π stacking interactions with aromatic residues in the hydrophobic core of Aβ, thereby destabilizing amyloidogenic conformations [13,14,15].

Competitive inhibition of Aβ42 self-assembly using short peptide fragments derived from the native Aβ sequence has also emerged as an attractive molecular design-based strategy. These short peptides can bind Aβ with high specificity and suppress the formation of toxic aggregates. We previously developed Aβ42 aggregation inhibitors based on the C-terminal region of Aβ42, the 42-residue isoform that is structurally and functionally distinct from the 40-residue Aβ40. The additional Ile41 and Ala42 residues confer markedly higher aggregation propensity and neurotoxicity [16]. One structural basis for this phenomenon is the stabilizing intermolecular hydrogen bond formed between the carboxylate side chain of Ala42 (–COO^−^) and the protonated ε-amino group of Lys28 (–NH_3_^+^), which reinforces parallel β-sheet packing during fibril maturation [17]. To competitively disrupt this interaction, we previously engineered peptide conjugates consisting of an Aβ42-derived address domain linked to an anchoring moiety such as caffeic acid or vitamin E. These hybrid constructs selectively bound Aβ42, interfered with key hydrogen-bonding motifs, inhibited β-sheet formation, and potently suppressed Aβ-induced neurotoxicity [18,19].

Numerous studies have sought to expand this pharmacophore concept by employing other partial sequences of Aβ, such as residues Aβ_16–22_ or Aβ_26–36_, both of which strongly interact with β-sheet interfaces and exhibit robust aggregation-inhibiting activity [20,21,22,23,24]. More recently, peptide analogs designed to target the two hydrophobic pharmacophores of Aβ, Aβ17–21 (LVFFA) and Aβ36–42 (VGGVVIA), have been shown to inhibit and even reverse Aβ fibrillization through a novel soft-aggregation mechanism, further demonstrating the therapeutic potential of pharmacophore-based peptide engineering [25]. To further enhance potency and metabolic stability, a variety of structural modification strategies have been applied to KLVFF- and LVFF-based peptides. These include backbone N-methylation to disrupt β-sheet propagation [26], cyclization to impose conformational constraints [27], and retro-inverso or D-amino acid substitution to improve resistance to proteolytic degradation [28]. In addition, terminal modifications and side-chain protection have been shown to modulate self-assembly behavior and strengthen interactions with amyloidogenic interfaces. Collectively, these approaches increase inhibitory efficacy by preorganizing key hydrophobic and aromatic residues, suppressing β-sheet formation, and stabilizing non-toxic off-pathway assemblies. Such chemically engineered peptide derivatives therefore represent a robust strategy for enhancing the anti-aggregation activity of Aβ-derived pharmacophores.

Building on these advances, we focused on rational modification of Phe19 and Phe20, two aromatic residues critically involved in π–π stacking within parallel β-sheet fibrils. During the early stages of aggregation, these phenylalanine residues align with counterparts in adjacent Aβ molecules, forming intermolecular aromatic stacking interactions that stabilize oligomer growth. To competitively disrupt this process while enhancing Aβ-binding affinity, we designed Aβ_16–21_ peptides in which Phe19 and Phe20 were substituted with L-3,4-dihydroxyphenylalanine (DOPA). The catechol moiety of DOPA confers superior π-stacking potential and hydrogen-bonding versatility. We hypothesized that DOPA-substituted peptides would preferentially associate with aggregation-prone regions of Aβ, diverting self-assembly toward nonproductive complexes and thereby attenuating fibril propagation.

To evaluate this hypothesis, we assessed the effects of DOPA-modified peptides on Aβ42 aggregation using thioflavin T (ThT) fluorescence assays, circular dichroism (CD) spectroscopy, and transmission electron microscopy (TEM). Furthermore, their cytoprotective effects against Aβ42-induced neurotoxicity were examined in human SH-SY5Y neuroblastoma cell lines. Collectively, our results demonstrate that the rational modulation of intermolecular aromatic interactions represents a powerful molecular design strategy for controlling Aβ42 self-assembly. These findings highlight the potential of DOPA-substituted Aβ-derived peptides as promising therapeutic candidates for mitigating the pathological progression of AD.

## 2. Results and Discussion

### 2.1. Design Strategy for Aβ42 Aggregation-Inhibiting Peptides

Aβ peptides readily assemble into intermolecular parallel β-sheet structures during aggregation, a process in which the phenyl rings of Phe19 and Phe20 align with equivalent residues on adjacent Aβ molecules to form stabilizing π–π stacking interactions [29,30]. These aromatic contacts significantly contribute to β-sheet stabilization and thereby facilitate fibril propagation. We hypothesized that the intrinsic aggregation-inhibiting activity of the Aβ partial sequence Aβ_16–20_ (KLVFF) originates from its ability to engage in Phe19- and Phe20-mediated aromatic interactions with high affinity, thereby competitively interfering with native intermolecular stacking motifs within full-length Aβ.

To enhance the Aβ-binding affinity beyond that achieved by native aromatic residues, we focused on replacing the phenyl side chains of Phe19 and Phe20 with catechol-containing moieties capable of forming stronger and more versatile π–π interactions. Catechol and other phenolic rings are known to interact robustly with aromatic residues in Aβ. Moreover, several polyphenolic antioxidants, such as epigallocatechin and resveratrol, have been shown to suppress Aβ42 aggregation through competitive π–π stacking with the phenyl rings of Phe19 and Phe20 [31]. These findings support the concept that disruption of aromatic packing within the hydrophobic core effectively perturbs β-sheet formation by diverting aggregation interfaces toward alternative binding partners.

**Figure 1:**
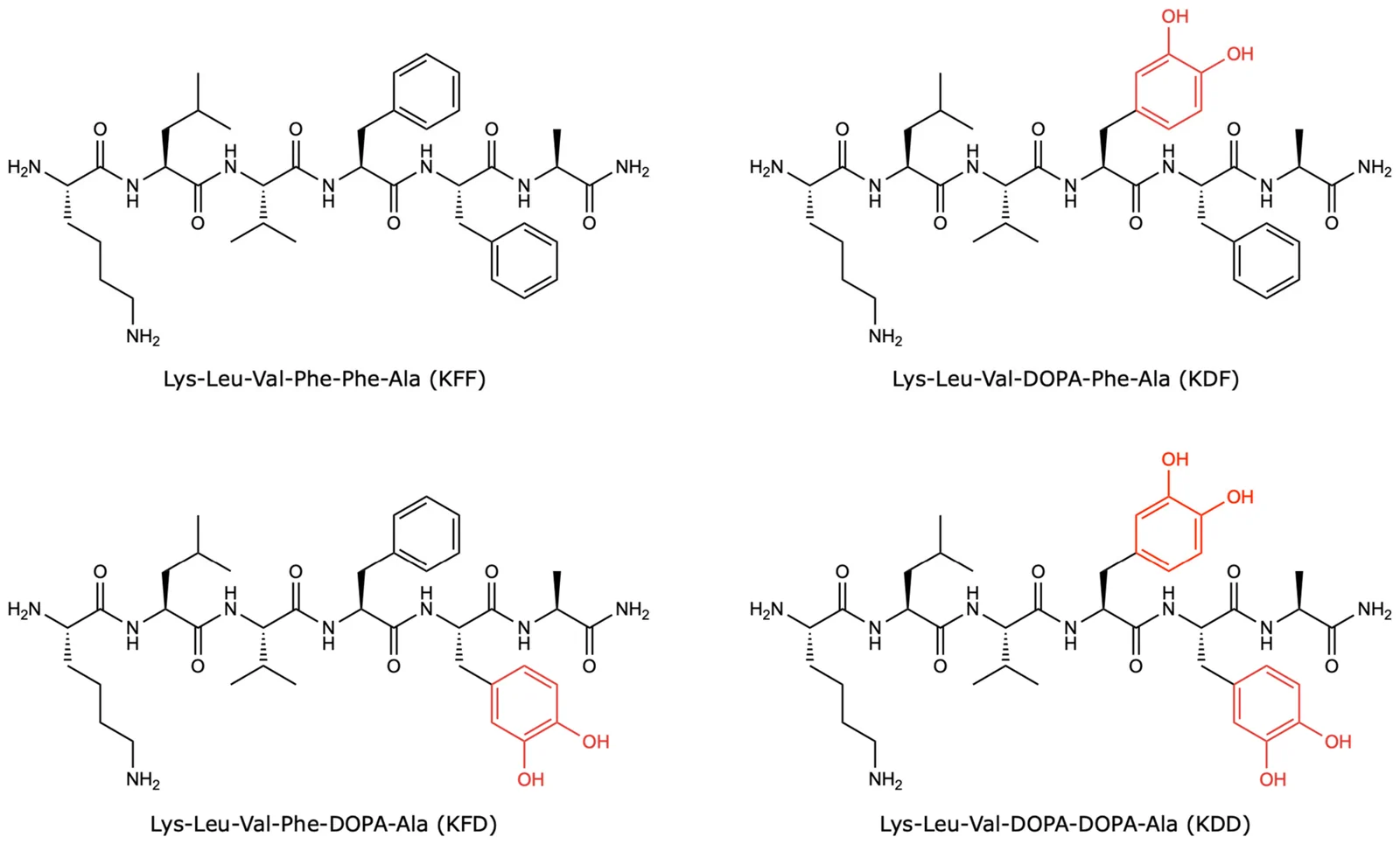
Molecular structures of the parent KLVFFA peptide and its DOPA-substituted derivatives. KFF (KLVFFA) is the parent peptide derived from Aβ_16–21_. KDF (KLVDpFA) and KFD (KLVFDpA) each contain a single DOPA substitution at Phe19 and Phe20, respectively, whereas KDD (KLVDpDpA) contains DOPA substitutions at both positions. Dp represents L-3,4-dihydroxyphenylalanine (DOPA).

Based on this rationale, we designed a series of peptide analogs derived from the recognition sequence KLVFFA (KFF), in which Phe19 or Phe20 was substituted with DOPA (Dp), a residue possessing a catechol group (Figure 1). Previous studies have reported aggregation inhibition using peptides of various lengths centered around Aβ_16–20_ (KLVFF), among which the core Aβ_16–20_ motif (KLVFF) was identified as crucial for aggregation [20,22,23,24]. In our approach, we selected this core region, specifically the most essential sequence, and extended it to Aβ_16–21_ (KLVFFA) to better capture the dynamics of its interaction with Aβ. To accurately facilitate interaction with the full-length Aβ, we then introduced DOPA substitutions at Phe19 or Phe20, creating the peptides KLVDpFA (KDF) and KLVFDpA (KFD). The doubly DOPA-substituted peptide, KLVDpDpA (KDD), was designed to incorporate DOPA at both aromatic sites. These derivatives maintain high target specificity while providing enhanced potential for competitive aromatic interactions with full-length Aβ. We anticipated that incorporating DOPA would reinforce noncanonical π–π stacking with phenylalanine residues of Aβ, thereby increasing binding affinity, promoting competitive occupation of aggregation-prone interfaces, and ultimately suppressing self-assembly more effectively than the unmodified KLVFFA sequence.

Collectively, this molecular design strategy exploits the aromatic packing dependencies of Aβ42 fibrillization and leverages catechol-mediated interactions to interfere with β-sheet stabilization. These peptide-based inhibitors are expected to bind competitively to aggregation hot spots, disrupt key intermolecular contacts, and robustly attenuate pathogenic Aβ42 aggregate formation.

### 2.2. Inhibition of Aβ42 Aggregation

**Figure 2:**
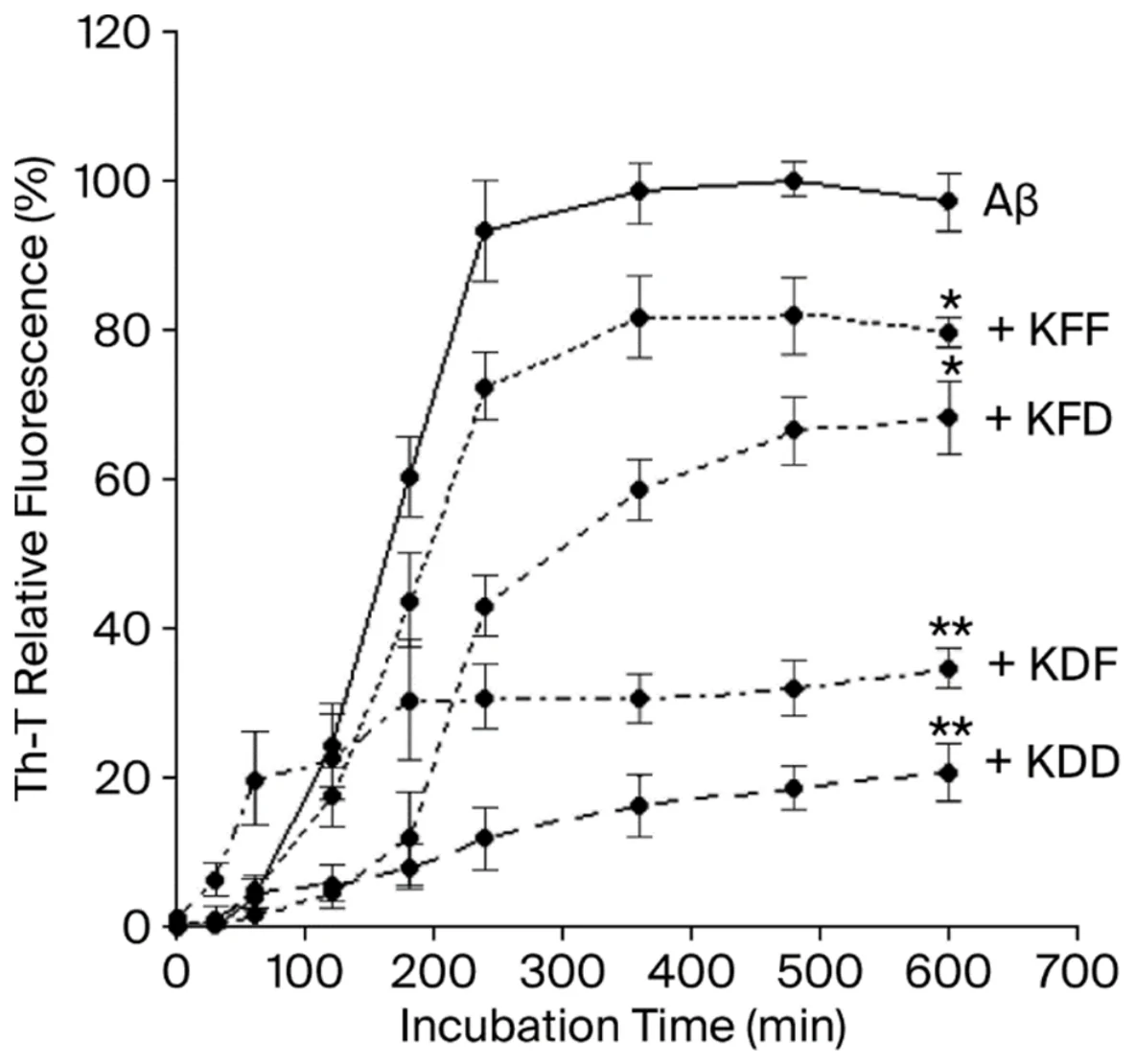
Inhibitory effects of KFF and DOPA-substituted peptides on Aβ42 aggregation kinetics monitored by thioflavin T (ThT) fluorescence. Aβ42 (20 µM) was incubated with or without peptides (20 µM) at 37 °C, and ThT fluorescence intensity was measured over time to monitor fibril formation. KFF is the parent KLVFFA peptide; KDF, KFD, and KDD contain DOPA substitutions at Phe19, Phe20, and both positions, respectively. Data are presented as the mean ± SD of three independent experiments (*n* = 3). Statistical comparisons were performed at 600 min using one-way ANOVA followed by Dunnett’s multiple comparisons test, with Aβ42 alone as the reference group. * *p* < 0.05 and ** *p* < 0.01 versus Aβ42 alone.

To evaluate the effects of DOPA substitution on Aβ42 aggregation, we employed a ThT fluorescence assay. ThT selectively binds to β-sheet-rich amyloid fibrils, resulting in a marked increase in fluorescence intensity; thus, the progressive formation of β-sheet structures can be monitored in real time [32]. In the presence of aggregation inhibitors, reduced β-sheet formation leads to diminished ThT binding and consequently lowers fluorescence signals. Using this assay, we assessed the inhibitory activities of the designed peptide analogs (Figure 2).

When 20 μM of Aβ42 was incubated without 20 μM of peptide, fluorescence intensity steadily increased over time, consistent with the expected progression of fibril formation. After approximately four hours, the fluorescence signal reached a plateau, indicating that the majority of Aβ42 had assembled into mature fibrillar structures. This plateau intensity was defined as 100% aggregation for quantitative comparison with peptide inhibitors added at equimolar concentrations. Consistent with previous studies demonstrating that KLVFFA can interfere with Aβ self-assembly by competitively binding to the hydrophobic core of Aβ [20,21,22,24], the parent peptide KFF exhibited measurable inhibitory activity, reducing the endpoint fluorescence to approximately 80% of that of the control. In contrast, substitution of either Phe19 or Phe20 with DOPA significantly enhanced anti-aggregation potency. The incorporation of DOPA at position 19 (KDF) suppressed fluorescence to approximately 30%, demonstrating a strong inhibitory effect; substitution at position 20 (KFD) also reduced fluorescence, albeit to a lesser extent than KDF (to approximately 60%). These findings suggest positional dependence in the contribution of aromatic interactions to early aggregation events, with Phe19 likely playing a more prominent role in stabilizing β-sheet stacking interfaces. When both phenylalanine residues were simultaneously replaced with DOPA (KDD), aggregation was further suppressed, with fluorescence intensity dramatically reduced to approximately 20%. This pronounced inhibition suggests that the cooperative action of two catechol moieties enhances competitive binding to aromatic hot spots within Aβ, which may interfere with the π–π stacking interactions involved in fibril growth.

Collectively, these results demonstrate that DOPA substitution at Phe19 and Phe20 effectively augments the anti-aggregation capability of the KLVFFA scaffold in a position-dependent manner and that dual substitution yields a synergistic enhancement of inhibitory activity.

### 2.3. CD Analysis of β-Sheet Formation

While the ThT fluorescence assay is widely used to monitor Aβ aggregation through the enhanced fluorescence of ThT bound to β-sheet-rich fibrils, it does not directly quantify the suppression of β-sheet secondary structure. Therefore, CD spectroscopy was used to more directly evaluate the effects of peptide inhibitors on β-sheet formation. Aggregated Aβ42 predominantly adopts a β-sheet structure characterized by a distinct negative band near 218 nm; thus, the intensity of this band serves as a quantitative indicator of β-sheet content during aggregation [33].

**Figure 3:**
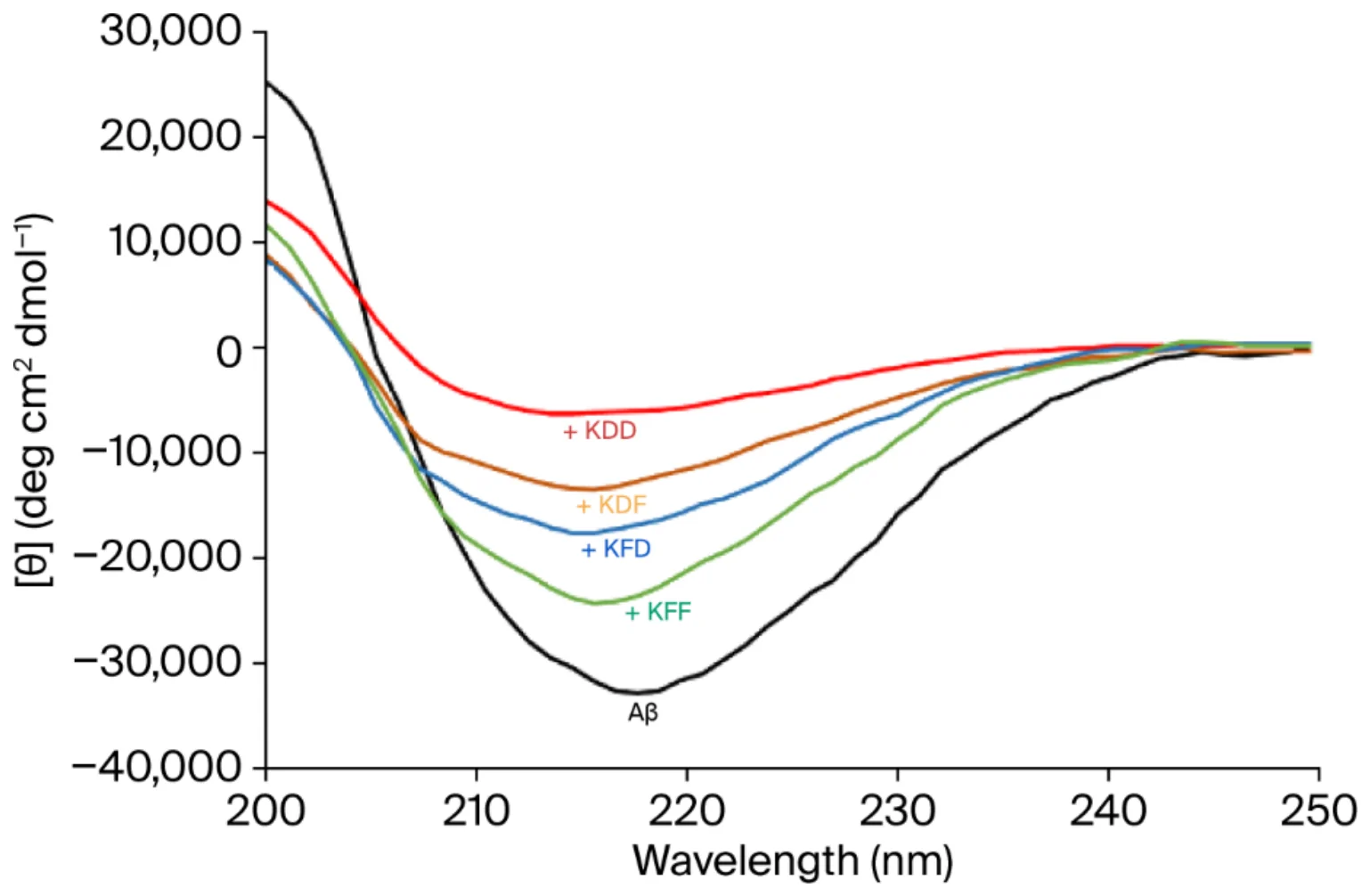
Effects of synthetic peptides on Aβ42 β-sheet formation, analyzed using CD measurements. CD spectra of Aβ42 (20 µM) were recorded after incubation at 37 °C for 4 h in the absence or presence of peptides (20 µM). Each CD spectrum represents the average of three measurements performed under the same experimental conditions. KFF is the parent KLVFFA peptide; KDF, KFD, and KDD contain DOPA substitutions at Phe19, Phe20, and both positions, respectively.

Mixtures containing 20 µM of Aβ42 and 20 µM of peptide were incubated at 37 °C for 4 h prior to CD spectral measurements. Peptide-free controls were prepared under identical conditions. In the peptide-free controls, aggregated Aβ42 exhibited a strong negative signal at approximately 218 nm, consistent with extensive β-sheet formation (Figure 3). Addition of the Aβ_16–21_ partial sequence (KFF), previously shown by ThT analysis to attenuate Aβ42 aggregation, reduced the 218 nm band intensity to around 75% of that of the control, indicating partial suppression of β-sheet assembly. Substitution of either Phe19 or Phe20 with DOPA further inhibited secondary structure formation. In the presence of the mono-substituted analogs KFD and KDF, CD intensity decreased to approximately 50% and 40% of the control, respectively. Notably, simultaneous replacement of both phenylalanine residues with DOPA in KDD yielded the most pronounced suppression, reducing the CD signal to approximately 20% of that of the control, indicative of substantial inhibition of β-sheet formation.

Overall, the degree of CD intensity reduction correlated closely with the inhibition profiles observed in the ThT fluorescence assay, supporting the interpretation that the observed spectral attenuation reflects decreased formation of β-sheet-rich fibrillar structures. These findings suggest that the enhanced inhibitory potency of DOPA-substituted peptides arises from their ability to effectively disrupt aromatic packing interactions critical for β-sheet assembly. The superior activity of KDF relative to KFD further implies positional sensitivity, with modification at Phe19 exerting a greater perturbative effect on fibril structure than Phe20 substitution.

### 2.4. TEM Analysis

Aggregation of Aβ42 proceeds through sequential structural transitions, in which soluble oligomeric intermediates evolve into extended fibrillar assemblies enriched in the β-sheet secondary structure. These fibrillar aggregates are believed to contribute to amyloid plaque formation in vivo and are strongly associated with neurotoxicity. The ThT fluorescence and CD spectroscopy results demonstrated that the designed peptides suppress β-sheet formation and inhibit fibril elongation. To further elucidate the morphological consequences of adding these peptides, TEM was employed.

**Figure 4:**
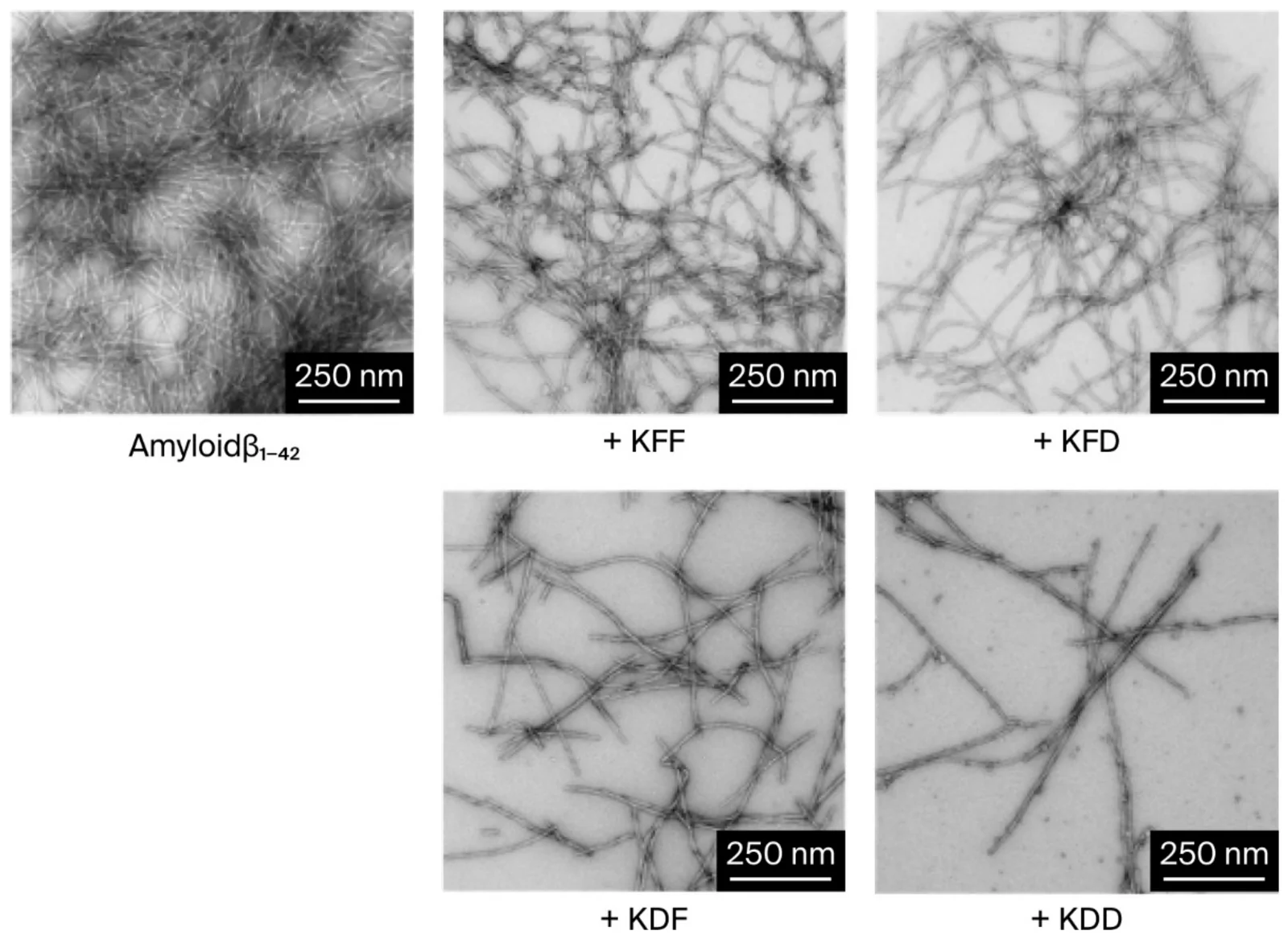
TEM analysis of Aβ42 fibril morphology in the presence of synthetic peptides. Aβ42 (10 µM) was incubated alone or with synthetic peptides (10 µM) at 37 °C for 24 h, followed by negative staining and transmission electron microscopy. KFF is the parent KLVFFA peptide; KDF, KFD, and KDD contain DOPA substitutions at Phe19, Phe20, and both positions, respectively.

Ten μM of Aβ42 was incubated in phosphate-buffered saline for 24 h in the presence or absence of peptide inhibitors prior to TEM imaging (Figure 4). In the absence of peptide inhibitors, abundant elongated fibrils were observed, which formed dense, mesh-like networks characteristic of mature amyloid assemblies. This morphology is consistent with extensive β-sheet organization and reflects the strong fibrillogenic propensity of Aβ42 under physiological ionic conditions.

Fibrillar structures were still detected after the addition of the parent peptide KFF. However, their abundance and network density were clearly reduced compared with the peptide-free control, consistent with biochemical analyses indicating that KFF partially interferes with fibril growth. More pronounced inhibitory effects were observed when the phenylalanine residues of KFF were substituted with DOPA. Both mono-substituted analogs, KDF and KFD, substantially suppressed fibril formation, with KDF exhibiting more pronounced effects congruent with its superior inhibition of β-sheet assembly. Strikingly, the doubly substituted analog KDD yielded only sparse, short, and poorly organized filaments, suggesting that formation of mature fibrillar aggregates was impeded.

The extent of fibril suppression observed via TEM correlated well with the reductions in ThT fluorescence intensity and attenuation of the 218 nm β-sheet band present in the CD spectra, providing orthogonal validation of peptide-mediated aggregation inhibition. Collectively, these results indicate that substituting phenylalanine with catechol-containing residues enhances the peptide scaffold’s ability to disrupt β-sheet stabilization, thereby preventing the hierarchical assembly of toxic fibrillar aggregates.

### 2.5. Inhibition of Aβ42-Induced Cytotoxicity

Aβ42 peptides exhibit potent neurotoxicity during self-assembly from soluble monomers to β-sheet-rich fibrillar aggregates. Soluble oligomeric intermediates formed at early aggregation stages are particularly deleterious, as they disrupt membrane integrity, dysregulate calcium ion influx, and enhance ROS generation, collectively provoking intracellular stress and, ultimately, cell death [7,34,35]. Consistent with these mechanistic insights, treatment of SH-SY5Y neuroblastoma cells with Aβ42 results in reduced cell viability and morphological deterioration, underscoring the pathological relevance of aggregation-driven cytotoxicity.

**Figure 5:**
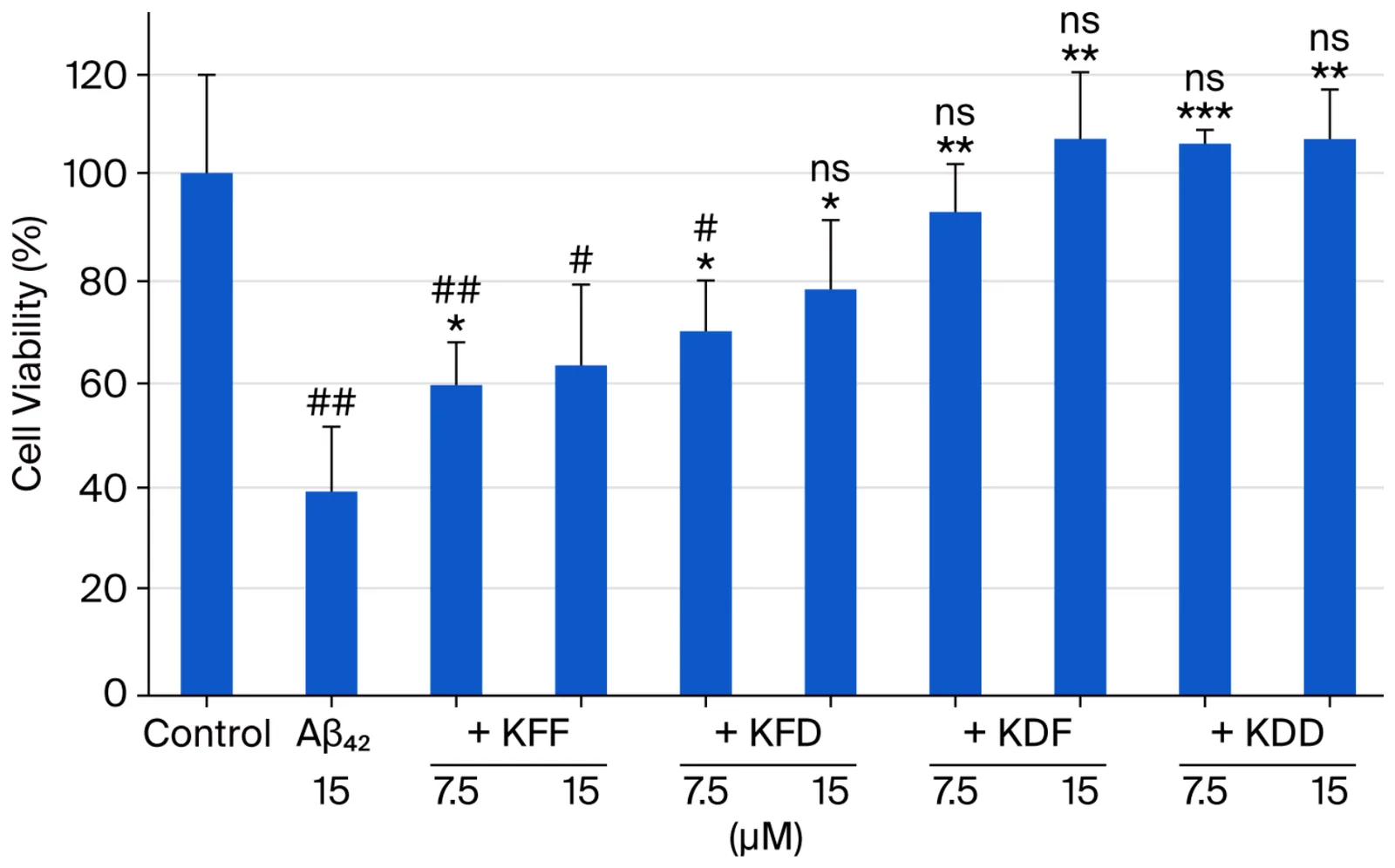
Protective effects of synthetic peptides against Aβ42-induced cytotoxicity in SH-SY5Y cells. SH-SY5Y cells were incubated with Aβ42 (15 µM) in the absence or presence of peptides (7.5 or 15 µM) at 37 °C for 48 h. Cell viability was assessed using the WST-8 assay. KFF is the parent KLVFFA peptide; KDF, KFD, and KDD contain DOPA substitutions at Phe19, Phe20, and both positions, respectively. Data are presented as the mean ± SD of three independent experiments (*n* = 3). Statistical significance was evaluated using one-way ANOVA followed by Dunnett’s multiple comparisons test, with Aβ42 alone or the untreated control group as the reference, as indicated. * *p* < 0.05, ** *p* < 0.01, and *** *p* < 0.001 versus Aβ42 alone; # *p* < 0.05 and ## *p* < 0.01 versus the untreated control group. ns indicates no statistically significant difference from the untreated control group (*p* ≥ 0.05).

To evaluate whether the DOPA-substituted peptides could mitigate Aβ42-induced neurotoxicity, SH-SY5Y cells were incubated with 15 μM of Aβ for 48 h in the absence or presence of peptide inhibitors. Importantly, in the absence of Aβ42, none of the DOPA-substituted peptides exhibited detectable cytotoxicity toward SH-SY5Y cells, indicating that the introduced catechol-containing peptides themselves do not induce measurable cellular toxicity under this study’s experimental conditions. Treatment with Aβ42 alone decreased cell viability to approximately 40%, confirming robust cytotoxicity in this model system (Figure 5). Co-incubation with the parent KFF peptide modestly attenuated Aβ42 toxicity, increasing viability to approximately 60%, which is consistent with its partial inhibition of aggregation.

In contrast, substitution of phenylalanine residues with DOPA conferred markedly enhanced, concentration-dependent protective effects. Among the mono-substituted analogs, KDF exhibited superior neuroprotection compared with KFD. At 7.5 μM of peptide, KFD increased viability to approximately 70%, whereas KDF restored viability to about 90%. Further increasing the KDF concentration to 15 μM nearly nullified cytotoxicity, restoring viability to baseline levels. Notably, dual substitution of both phenylalanine residues (KDD) yielded the strongest protective effect, restoring cell viability to nearly 100% at 7.5 μM.

These results demonstrate that incorporation of catechol-containing residues substantially enhances the peptide scaffold’s ability to counteract Aβ42-induced neurotoxicity. This enhanced cytoprotective effect is consistent with the inhibition of β-sheet formation and fibrillization observed via ThT fluorescence, CD spectroscopy, and TEM, suggesting that suppression of Aβ42 aggregation contributes to the observed protective effect against cellular damage. In addition to inhibiting Aβ42 aggregation, the catechol moieties introduced by DOPA substitution may also contribute to cytoprotection through their potential antioxidant properties. Because catechol-containing compounds can exhibit radical-scavenging activity, DOPA-substituted peptides may attenuate oxidative stress associated with Aβ42-induced cellular damage. However, neither the antioxidant activities of these peptides nor their effects on intracellular reactive oxygen species were directly evaluated in the present study. Further investigations are therefore needed to determine whether antioxidant mechanisms contribute to the observed cytoprotective effects.

### 2.6. Binding Free Energy Analysis

In this study, substitution of phenylalanine residues within the KFF sequence with DOPA was hypothesized to modify aromatic and other noncovalent interactions with Aβ42, thereby influencing β-sheet formation and fibrillization. The experimental results demonstrated that DOPA substitution enhanced the aggregation-inhibiting activity of the peptide scaffold, although the magnitude of inhibition depended on the substitution position and the number of DOPA residues. These effects were also associated with the attenuation of Aβ42-induced neurotoxicity in cultured neuronal cells. To investigate the molecular basis of these effects, a structure-based computational analysis was performed using the experimentally determined Aβ42 amyloid fibril model reported by Xiao et al. (PDB ID: 2MXU) [17]. This solid-state NMR-derived structure was selected as a representative fibril conformation to evaluate interactions between the peptides and the Lys16–Ala21 region of Aβ42. Following structural optimization, binding free energies were estimated using the GBVI/WSA method implemented in MOE.

**Figure 6:**
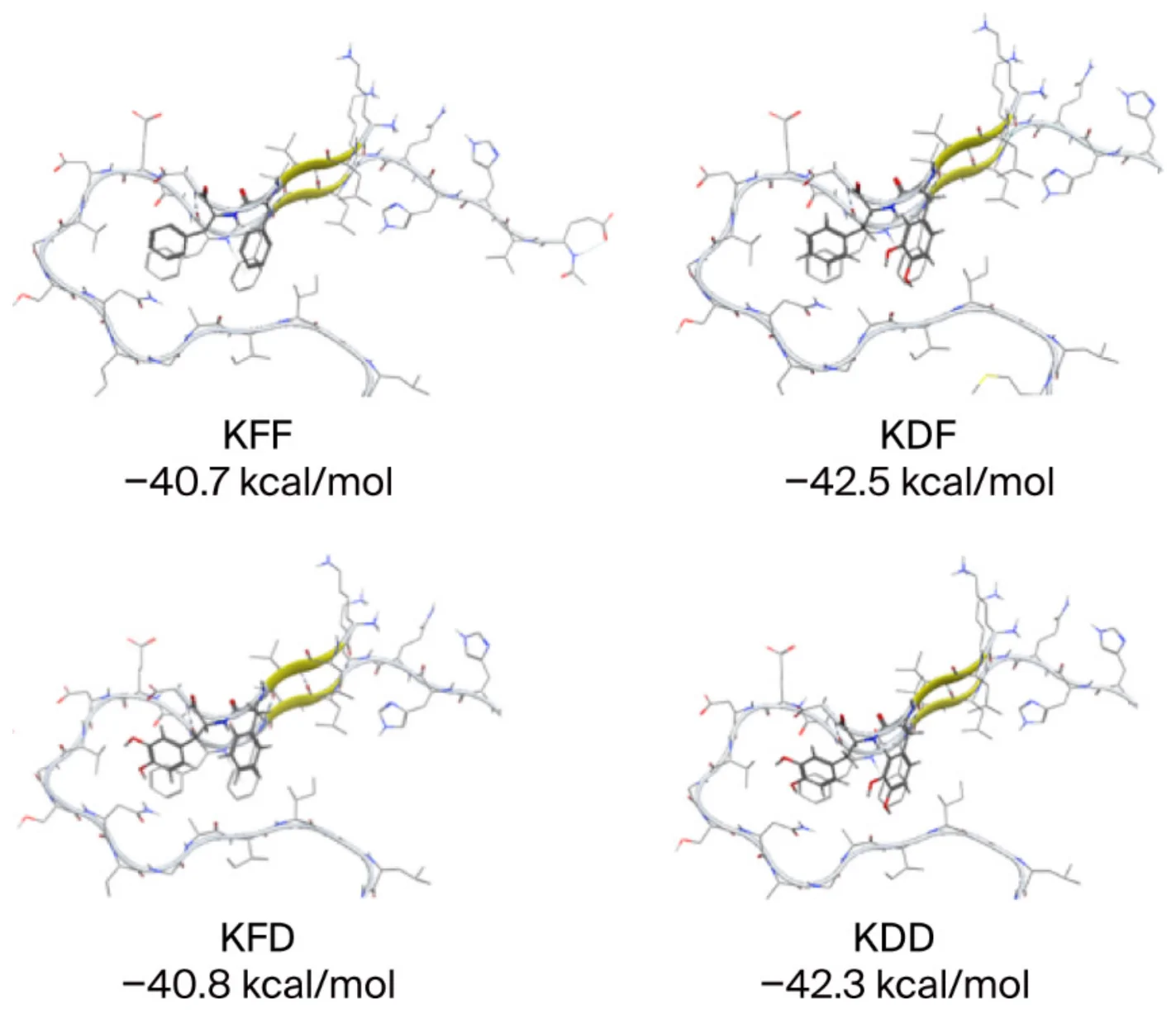
Structure-based estimates of binding free energies for KLVFFA and DOPA-substituted peptides interacting with the Aβ42 fibril interface. Peptide–Aβ42 complexes were constructed using the solid-state NMR-derived Aβ42 fibril structure (PDB ID: 2MXU) as the structural template. Binding free energies were estimated using the GBVI/WSA method implemented in MOE with the Amber10 force field following structural optimization. The calculated values provide a comparative assessment of the energetic effects of DOPA substitution within the selected fibril model.

The calculated binding free energies were −40.7, −40.8, −42.5, and −42.3 kcal mol^−1^ for KFF, KFD, KDF, and KDD, respectively (Figure 6). Substitution of Phe20 with DOPA (KFD) resulted in little change in the calculated energy relative to the parent KFF peptide. In contrast, substitution at Phe19 (KDF) yielded a more favorable calculated energy, and a similar result was obtained for the doubly substituted peptide KDD. These results suggest that the position of DOPA substitution can influence the calculated energetic characteristics of peptide interactions with the Aβ42 fibril interface within the structural model employed. The calculated energies of KDF and KDD were nearly identical, differing by only 0.2 kcal mol^−1^. Furthermore, the differences among the calculated energies were relatively small and should be interpreted cautiously in view of the inherent uncertainties associated with the computational method. Thus, these results do not provide sufficient evidence to establish reliable quantitative differences in binding affinity among the peptides but suggest that DOPA substitution may modify their interactions with the selected Aβ42 fibril structure.

Interestingly, although KDF and KDD exhibited comparable calculated binding free energies, KDD showed greater aggregation-inhibiting activity in the experimental assays. This discrepancy suggests that the aggregation-inhibiting activity of these peptides cannot be explained solely by the binding free energies estimated from a single optimized Aβ42 fibril structure. The presence of two catechol moieties in KDD may increase the potential for hydrogen bonding and aromatic interactions, which could facilitate interactions with different aggregation-prone conformations of Aβ42. Such interactions, which are not fully captured by the present computational model, may contribute to the enhanced inhibitory activity of KDD. However, the individual contributions of these interactions cannot be determined from the calculated binding free energies alone. This study’s computational analysis was intended to provide a comparative assessment of the energetic effects of DOPA substitution using a common, experimentally derived Aβ42 fibril structure. Although the GBVI/WSA method incorporates an approximate treatment of solvation, the calculations were based on optimized structures and did not explicitly account for conformational ensembles, solvent dynamics, or alternative protonation states of ionizable residues. These factors may substantially influence peptide–Aβ42 interactions, particularly during the dynamic process of amyloid aggregation. Accordingly, the calculated energies should be regarded as model-dependent comparative estimates rather than definitive measures of binding affinity under physiological conditions.

Previous studies suggested that catechol-containing compounds can inhibit Aβ aggregation through oxidative conversion to quinones, followed by covalent reactions such as Michael addition [36]. In addition to such mechanisms, our findings suggest that catechol-containing peptides may influence Aβ aggregation through noncovalent interactions, potentially involving π–π stacking with phenylalanine residues. However, our calculations do not directly establish the occurrence or relative contribution of π–π stacking interactions, and further structural and mechanistic investigations are required to clarify their role. A recent study also suggested that the KLVFFA fragment may not play a dominant role in the formation of early Aβ oligomer complexes [37]. Our findings do not necessarily contradict this observation because DOPA substitution may alter the interaction properties of the parent peptide sequence and potentially enable recognition of Aβ conformations that differ from those preferentially recognized by the unmodified peptide. Nevertheless, the specific aggregation intermediates targeted by the DOPA-substituted peptides remain to be identified.

Taken together, the experimental and computational results suggest that DOPA substitution modulates peptide–Aβ42 interactions in a position-dependent manner, but the resulting aggregation-inhibiting activity cannot be predicted solely from binding energies calculated using a mature fibril structure. These findings highlight the importance of considering both the structural characteristics of the peptide derivatives and the conformational heterogeneity of Aβ42 during aggregation. Further investigations, including molecular dynamics simulations of the peptides interacting with different Aβ42 conformational states, are underway to clarify the molecular mechanisms underlying their aggregation-inhibiting activities.

## 3. Materials and Methods

### 3.1. Methods

The control peptide (KLVFFA) and DOPA-substituted analogs (KLVFDpA, KLVDpFA, and KLVDpDpA) were synthesized by standard Fmoc-based solid-phase peptide synthesis. Rink amide PEG resin, Fmoc-protected amino acids, and Fmoc-DOPA(3,4-diOxProp)-OH were purchased from Watanabe Chemical Industries, Ltd. (Hiroshima, Japan). Amino acid couplings were performed in N,N-dimethylformamide (DMF) using HBTU/HOBt as coupling reagents in the presence of N,N-diisopropylethylamine, with each coupling step lasting 1 h. Fmoc deprotection was carried out using 20% piperidine in DMF. After the completion of chain assembly, the peptides were cleaved from the resin and globally deprotected with a trifluoroacetic acid/triisopropylsilane/H_2_O mixture (95:2.5:2.5, v/v/v) for 3 h. The reaction mixture was filtered, and the filtrate was concentrated under a nitrogen stream. The crude peptides were precipitated with cold diethyl ether, collected by centrifugation, washed with ether, and dried under reduced pressure. Purification was performed by preparative reverse-phase high-performance liquid chromatography (RP-HPLC) using a C18 column. The purity of the isolated peptides (>98%) was confirmed by analytical RP-HPLC. High-resolution mass spectrometry (HRMS) analysis confirmed the molecular masses of all peptides as follows:

HRMS (ESI) m/z [M+H]^+^ calcd for KLVFFA (C_38_H_59_N_8_O_6_), 723.4552; found, 723.4474; calcd for KLVFDpA (C_38_H_59_N_8_O_8_), 755.4450; found, 755.4372; calcd for KLVDpFA (C_38_H_59_N_8_O_8_), 755.4450; found, 755.4367; calcd for KLVDpDpA (C_38_H_58_N_8_O_10_), 787.4349; found, 787.4301.

### 3.2. Aβ42 Pretreatment

To minimize pre-aggregation, Aβ42 (0.25 mg) was dissolved in hexafluoro-2-propanol (HFIP, 0.20 mL) to generate a monomeric stock. HFIP was removed by vacuum centrifugal evaporation to yield a thin peptide film, which was stored until use. For each experiment (ThT fluorescence, CD spectroscopy, TEM, and cell viability assays), the HFIP film was freshly dissolved in the appropriate buffer immediately before assays. The concentration of Aβ42 for each assay was carefully optimized to clearly and sensitively evaluate the differences in the inhibitory effects among the peptides under the specific technical constraints of each experimental system. Accordingly, the experiments described in this study evaluate the inhibition of Aβ42 aggregation from freshly prepared peptide solutions rather than the disruption of pre-formed oligomers or mature fibrils.

### 3.3. ThT Fluorescence Assay

Aβ42 was dissolved in 0.3% ammonium hydroxide to 1 mM with brief sonication. Peptides were prepared in DMF at 3 mM; the final concentration of DMF in the assay mixture was 0.1% (*v*/*v*). Vehicle controls containing the same concentration of DMF without the peptide were included in all experiments to confirm the absence of solvent interference. For each condition, a master mix was assembled in the following order: 25 µL of 0.2 M phosphate buffer (pH 7.4), 64 µL of Milli-Q water, 5 µL of 2.0 M NaCl, 2 µL of 2 mM peptide stock (or DMF for controls), and 100 µL of 20 µM ThT in glycine–NaOH buffer (pH 9.0). A total of 196 µL of the master mix and 4 µL of the 1mM Aβ42 solution were added to each well of a black, non-binding, 96-well plate (µClear; Greiner Bio-One, Frickenhausen, Germany) and incubated at 37 °C. Fluorescence was recorded at 0, 0.5, 1, 2, 3, 4, 6, 8, and 10 h using a Varioskan LUX microplate reader (Thermo Fisher Scientific, Waltham, MA, USA) with λ_ex 450 nm and λ_em 485 nm. Plates were shaken for 15 s immediately before each read. Data for each condition were obtained from three independent experiments. Note: In this assay system, even when compounds were added after 6 h of incubation following fibril formation, no reduction in fluorescence was observed. This indicates that the decrease in fluorescence seen in earlier measurements reflects inhibition of fibril formation by the compounds rather than a direct effect on ThT fluorescence itself. Control experiments confirmed that the DOPA-substituted peptides alone did not generate detectable ThT fluorescence under the assay conditions, indicating that the peptides themselves neither formed ThT-positive assemblies nor contributed to the observed fluorescence signals.

### 3.4. CD Spectroscopy

Aβ42’s secondary structure was evaluated by CD spectroscopy. Aβ was dissolved in 0.3% NH_4_OH to 200 µM with brief sonication, and peptides were prepared in acetonitrile at 100 µM. For each measurement, samples were prepared in microcentrifuge tubes in the following order: 100 µL of 0.2 M phosphate buffer (pH 7.4), 230 µL of Milli-Q water, 20 µL of 2.0 M NaCl, 100 µL of 100 µM peptide solution (or acetonitrile for peptide-free controls), and 50 µL of 200 µM Aβ42 solution. They were then incubated at 37 °C for 4 h. The final concentration of acetonitrile was kept at 20% (*v*/*v*) to avoid background absorbance in the far-UV region. After incubation, an aliquot was transferred to a 0.1 mm pathlength quartz cuvette, and spectra were recorded from 200 to 250 nm using a J-720W spectropolarimeter (JASCO Corporation, Tokyo, Japan). The spectrum of the corresponding vehicle (buffer/water/NaCl with matching solvent) was subtracted from all datasets. Furthermore, to fully correct for any peptide-specific contributions and potential signal changes during incubation, the spectrum of each peptide solution alone, incubated under identical conditions (37 °C for 4 h), was subtracted from the respective mixture spectrum. For each experimental condition, CD spectra were recorded three times, and the averaged spectra were used for analysis and graphical presentation.

### 3.5. TEM

Aβ42 was dissolved in 0.3% ammonium hydroxide to 500 µM with brief sonication. Peptides were prepared in DMF at 1 mM; the final concentration of DMF in the assay mixture was 0.1% (*v*/*v*). For each condition, a master mix was prepared as follows: 50 µL of 0.2 M phosphate buffer (pH 7.4), 134 µL of Milli-Q water, 10 µL of 2.0 M NaCl, 2 µL of 1mM peptide stock (or DMF for controls). In total, 196 µL of the master mix and 4 µL of the 500 µM Aβ42 solution were combined in a microcentrifuge tube and incubated at 37 °C for 24 h. After incubation, the samples were gently mixed and, when indicated, centrifuged at 4 °C for 10 min. Aliquots (5–10 µL) were applied to carbon-coated copper grids and allowed to adsorb for 2–3 min. Excess liquid was blotted, and the grids were negative-stained with 2% (*w*/*v*) uranyl acetate for 6 min, followed by a 1 min rinse with distilled water. The grids were air-dried overnight and imaged on a Hitachi H-7600 transmission electron microscope (Hitachi High-Tech Corporation, Tokyo, Japan) operating at 80 kV. Images were acquired under identical settings across conditions.

### 3.6. Cell Culture

The human neuroblastoma cell line SH-SY5Y was obtained from the American Type Culture Collection (ATCC, catalog number CRL-2266). The cells were maintained in a 1:1 (*v*/*v*) mixture of Dulbecco’s modified Eagle’s medium and Ham’s F-12 (DMEM/F-12) supplemented with 10% fetal bovine serum (FBS) and penicillin–streptomycin (50 U/mL and 50 µg/mL, respectively). These cells were cultured at 37 °C in a humidified incubator under 5% CO_2_.

### 3.7. Aβ42-Induced Cytotoxicity and Peptide Protection Assay

Peptides were dissolved in dimethyl sulfoxide (DMSO); the final concentration of DMSO in the culture medium was 0.1% (*v*/*v*), at which no inherent cytotoxicity was observed. Cells treated with the same concentration of DMSO served as the vehicle control. Vehicle controls containing an equivalent amount of DMSO were included and showed no effect on viability. SH-SY5Y cells were seeded at 1.0 × 10^4^ cells/100 µL/well in type I collagen-coated black 96-well plates (Nippi, Tokyo, Japan) and allowed to adhere for 72 h. The culture medium was then replaced with phenol red-free DMEM/F-12 containing 2% FBS, 30 U/mL penicillin, and 30 µg/mL streptomycin. Test peptides (at the indicated concentrations) were added together with Aβ42 (stock 150 µM in 0.02% NH_4_OH, yielding a final Aβ42 concentration of 15 µM). After incubation for 48 h at 37 °C under 5% CO_2_, cell viability was quantified using WST-8 assay (Dojindo, Kumamoto, Japan) according to the manufacturer’s protocol. Briefly, 10 µL of WST-8 reagent was added per well, and plates were incubated for 4 h before absorbance was measured at 450 nm (reference 655 nm) using a GloMax^®^ Multi microplate reader (Promega Corporation, Madison, WI, USA). Data were normalized to untreated controls, with appropriate vehicle controls included in all experiments.

### 3.8. Computational Modeling and Binding Free Energy Calculations

Binding affinities between Aβ42 fibrils and the partial peptide variants were evaluated using a computational approach based on a previously reported structural model (PDB ID: 2MXU), which comprises 12 stacked Aβ units representing the 1-fold polymorph determined by solid-state NMR [17]. For accurate estimation of intermolecular interactions with the KLVFF motif, the central Aβ molecule (6th or 7th unit) was selected from model 1 to avoid edge effects during energy minimization. Peptide variants were constructed by substituting one or both phenylalanine residues in KLVFF with DOPA, resulting in the following sequences: KFF (unmodified), KDF (Phe19 → DOPA), KFD (Phe20 → DOPA), and KDD (both Phe residues → DOPA). Initial binding poses were generated by aligning the KLVFF motif with the Lys16–Ala21 region of the selected Aβ fibril. Geometry optimization and free energy calculations were conducted using MOE, version 2020.09(Chemical Computing Group ULC, Montréal, QC, Canada) with the Amber10:EHT force field. All atoms were allowed to move freely during optimization under the Born solvation model, while the Aβ backbone was constrained to preserve the fibril structure. A force tolerance threshold was set to 0.1 RMS kcal·mol^−1^·Å^−2^. The dielectric constants were set to 1 and 80 for the interior and exterior of each complex, respectively. Binding free energies were calculated using the GBVI/WSA method implemented in MOE. A comparative analysis of the four peptide variants provided insight into the effects of DOPA substitution on interaction strength with the Aβ fibril, particularly within the central stacking interface.

## 4. Conclusions

In this study, we designed and synthesized a series of peptide derivatives in which Phe19 and/or Phe20 of the Aβ-derived KLVFFA sequence were replaced with catechol-containing DOPA residues. ThT fluorescence assays, CD spectroscopy, and TEM collectively demonstrated that DOPA substitution suppressed Aβ42 aggregation and β-sheet-rich fibril formation, with the inhibitory effects dependent on the position and number of DOPA substitutions. In particular, substitution at Phe19 resulted in greater inhibitory activity than substitution at Phe20, whereas the doubly substituted peptide KDD exhibited the strongest overall anti-aggregation effect. Structure-based computational analysis further suggested that DOPA substitution at Phe19 favorably alters peptide interactions with the Aβ42 fibril interface. In addition, the DOPA-substituted peptides significantly attenuated Aβ42-induced reductions in SH-SY5Y cell viability, with KDF and KDD showing pronounced cytoprotective effects. These findings support the potential of DOPA substitution as a molecular design strategy for developing peptide-based inhibitors of Aβ42 aggregation and associated cellular toxicity. Further mechanistic investigations and in vivo evaluations are warranted to assess their therapeutic potential for Alzheimer’s disease.

## Data Availability

The data presented in this study are available in the article.

## Abbreviations

Aβ	amyloid-β
AD	Alzheimer’s disease
CD	circular dichroism
DMF	N,N-dimethylformamide
DMSO	dimethyl sulfoxide
DOPA	L-3,4-dihydroxyphenylalanine
HRMS	High-resolution mass spectrometry
KDD	KLVDpDpA
KDF	KLVDpFA
KFD	KLVFDpA
KFF	KLVFFA
NMR	nuclear magnetic resonance
RP-HPLC	reverse-phase high-performance liquid chromatography
TEM	transmission electron microscopy
ThT	thioflavin T

## References

[1] Prince M., Bryce R., Albanese E., Wimo A., Ribeiro W., Ferri C.P. (2013). The global prevalence of dementia: A systematic review and metaanalysis. Alzheimer’s Dement..

[2] DeTure M.A., Dickson D.W. (2019). The neuropathological diagnosis of Alzheimer’s disease. Mol. Neurodegener..

[3] Breijyeh Z., Karaman R. (2020). Comprehensive Review on Alzheimer’s Disease: Causes and Treatment. Molecules.

[4] Selkoe D.J., Hardy J. (2016). The amyloid hypothesis of Alzheimer’s disease at 25 years. EMBO Mol. Med..

[5] Chen G.F., Xu T.H., Yan Y., Zhou Y.R., Jiang Y., Melcher K., Xu H.E. (2017). Amyloid beta: Structure, biology and structure-based therapeutic development. Acta Pharmacol. Sin..

[6] Huang Y.R., Liu R.T. (2020). The Toxicity and Polymorphism of beta-Amyloid Oligomers. Int. J. Mol. Sci..

[7] Sengupta U., Nilson A.N., Kayed R. (2016). The Role of Amyloid-beta Oligomers in Toxicity, Propagation, and Immunotherapy. EBioMedicine.

[8] Zhao L.N., Long H.W., Mu Y., Chew L.Y. (2012). The toxicity of amyloid beta oligomers. Int. J. Mol. Sci..

[9] Kim B.H., Kim S., Nam Y., Park Y.H., Shin S.M., Moon M. (2025). Second-generation anti-amyloid monoclonal antibodies for Alzheimer’s disease: Current landscape and future perspectives. Transl. Neurodegener..

[10] Cummings J., Osse A.M.L., Cammann D., Powell J., Chen J. (2024). Anti-Amyloid Monoclonal Antibodies for the Treatment of Alzheimer’s Disease. BioDrugs.

[11] Ghadami S.A., Chia S., Ruggeri F.S., Meisl G., Bemporad F., Habchi J., Cascella R., Dobson C.M., Vendruscolo M., Knowles T.P.J. (2020). Transthyretin Inhibits Primary and Secondary Nucleations of Amyloid-beta Peptide Aggregation and Reduces the Toxicity of Its Oligomers. Biomacromolecules.

[12] Adwan L., Subaiea G.M., Zawia N.H. (2014). Tolfenamic acid downregulates BACE1 and protects against lead-induced upregulation of Alzheimer’s disease related biomarkers. Neuropharmacology.

[13] Pagano K., Tomaselli S., Molinari H., Ragona L. (2020). Natural Compounds as Inhibitors of Abeta Peptide Aggregation: Chemical Requirements and Molecular Mechanisms. Front. Neurosci..

[14] Doytchinova I., Atanasova M., Salamanova E., Ivanov S., Dimitrov I. (2020). Curcumin Inhibits the Primary Nucleation of Amyloid-beta Peptide: A Molecular Dynamic Study. Biomolecules.

[15] Porat Y., Abramowitz A., Gazit E. (2006). Inhibition of amyloid fibril formation by polyphenols: Structural similarity and aromatic interactions as a common inhibition mechanism. Chem. Biol. Drug Des..

[16] Benilova I., Karran E., De Strooper B. (2012). The toxic Abeta oligomer and Alzheimer’s disease: An emperor in need of clothes. Nat. Neurosci..

[17] Xiao Y., Ma B., McElheny D., Parthasarathy S., Long F., Hoshi M., Nussinov R., Ishii Y. (2015). Abeta(1-42) fibril structure illuminates self-recognition and replication of amyloid in Alzheimer’s disease. Nat. Struct. Mol. Biol..

[18] Arai T., Ohno A., Kazunori M., Kakizawa T., Kuwata H., Ozawa T., Shibanuma M., Hara S., Ishida S., Kurihara M. (2016). Design, synthesis, and evaluation of Trolox-conjugated amyloid-beta C-terminal peptides for therapeutic intervention in an in vitro model of Alzheimer’s disease. Bioorg. Med. Chem..

[19] Arai T., Ohno A., Mori K., Kuwata H., Mizuno M., Imai K., Hara S., Shibanuma M., Kurihara M., Miyata N. (2016). Inhibition of amyloid fibril formation and cytotoxicity by caffeic acid-conjugated amyloid-beta C-terminal peptides. Bioorg. Med. Chem. Lett..

[20] Abedin F., Kandel N., Tatulian S.A. (2021). Effects of Abeta-derived peptide fragments on fibrillogenesis of Abeta. Sci. Rep..

[21] Itoh S.G., Okumura H. (2021). Promotion and Inhibition of Amyloid-beta Peptide Aggregation: Molecular Dynamics Studies. Int. J. Mol. Sci..

[22] Lu J., Cao Q., Wang C., Zheng J., Luo F., Xie J., Li Y., Ma X., He L., Eisenberg D. (2019). Structure-Based Peptide Inhibitor Design of Amyloid-beta Aggregation. Front. Mol. Neurosci..

[23] Tjernberg L.O., Lilliehook C., Callaway D.J., Naslund J., Hahne S., Thyberg J., Terenius L., Nordstedt C. (1997). Controlling amyloid beta-peptide fibril formation with protease-stable ligands. J. Biol. Chem..

[24] Tjernberg L.O., Naslund J., Lindqvist F., Johansson J., Karlstrom A.R., Thyberg J., Terenius L., Nordstedt C. (1996). Arrest of beta-amyloid fibril formation by a pentapeptide ligand. J. Biol. Chem..

[25] Liu S., Peng F., Liu J., Wu W., He L., Cao H., Liu C., Qiu F., Zhang W. (2025). Peptides with Soft-Aggregation Behavior Inhibit and Reverse Amyloid-beta Fibrillization. Small.

[26] Kokkoni N., Stott K., Amijee H., Mason J.M., Doig A.J. (2006). N-Methylated peptide inhibitors of beta-amyloid aggregation and toxicity. Optimization of the inhibitor structure. Biochemistry.

[27] Arai T., Sasaki D., Araya T., Sato T., Sohma Y., Kanai M. (2014). A cyclic KLVFF-derived peptide aggregation inhibitor induces the formation of less-toxic off-pathway amyloid-beta oligomers. Chembiochem.

[28] Taylor M., Moore S., Mayes J., Parkin E., Beeg M., Canovi M., Gobbi M., Mann D.M., Allsop D. (2010). Development of a proteolytically stable retro-inverso peptide inhibitor of beta-amyloid oligomerization as a potential novel treatment for Alzheimer’s disease. Biochemistry.

[29] Stankovic I.M., Niu S., Hall M.B., Zaric S.D. (2020). Role of aromatic amino acids in amyloid self-assembly. Int. J. Biol. Macromol..

[30] Kumar J., Namsechi R., Sim V.L. (2015). Structure-Based Peptide Design to Modulate Amyloid Beta Aggregation and Reduce Cytotoxicity. PLoS ONE.

[31] Li F., Zhan C., Dong X., Wei G. (2021). Molecular mechanisms of resveratrol and EGCG in the inhibition of Abeta(42) aggregation and disruption of Abeta(42) protofibril: Similarities and differences. Phys. Chem. Chem. Phys..

[32] Biancalana M., Koide S. (2010). Molecular mechanism of Thioflavin-T binding to amyloid fibrils. Biochim. Biophys. Acta.

[33] Greenfield N.J. (2006). Using circular dichroism spectra to estimate protein secondary structure. Nat. Protoc..

[34] Kawahara M., Ohtsuka I., Yokoyama S., Kato-Negishi M., Sadakane Y. (2011). Membrane Incorporation, Channel Formation, and Disruption of Calcium Homeostasis by Alzheimer’s beta-Amyloid Protein. Int. J. Alzheimer’s Dis..

[35] Demuro A., Mina E., Kayed R., Milton S.C., Parker I., Glabe C.G. (2005). Calcium dysregulation and membrane disruption as a ubiquitous neurotoxic mechanism of soluble amyloid oligomers. J. Biol. Chem..

[36] Sato M., Murakami K., Uno M., Nakagawa Y., Katayama S., Akagi K., Masuda Y., Takegoshi K., Irie K. (2013). Site-specific inhibitory mechanism for amyloid beta42 aggregation by catechol-type flavonoids targeting the Lys residues. J. Biol. Chem..

[37] Lee H.T., Chang H.W., Lin Y.T., Huang S.J., Wu M.H., Tsai C.S., Tu L.H., Lee M.C., Chen Y.R., Chan J.C.C. (2025). Oligomeric Protein Complexes Formed by Beta Amyloid Peptides and Their Molecular Associates. Chemistry.

